# Supplementary material for: PHF6 promotes non‐homologous end joining and G2 checkpoint recovery
Source: EMBO Rep. 2019 Nov 29;21(1):e48460. doi: 10.15252/embr.201948460 (PMC6944915; doi:10.15252/embr.201948460)
Supplement: Supplementary file 1 — Expanded View Figures PDF [file EMBR-21-e48460-s001.pdf]

Expanded View Figures

Figure EV1. Effect of candidate proteins on viability in response to IR.

A Positive control for Fig 2D. U2OS cells were mock depleted or depleted for Luc or PPM1D. Next, cells were synchronized in G2, irradiated, and fixed for immunofluorescence for γH2AX after 0, 2, and 24 h. Scale bar is 20 μm. From one experiment, four separate images with a total of at least 100 cells were analyzed for each knockdown. The quantification shows the number of γH2AX foci per cell. In the box plots, the median with the first and third quartile is indicated, and the whiskers are drawn down to the 10th percentile and up to the 90th.

B Positive control for Fig 2E. U2OS cells were mock depleted or depleted for Luc or Artemis. Next, cells were synchronized in G2, irradiated, and fixed for immunofluorescence for 53BP1 after 0, 2, and 24 h. Scale bar is 20 μm. From one experiment, four separate images with a total of at least 100 cells were analyzed for each knockdown. The quantification shows the number of 53BP1 foci per cell. In the box plots, the median with the first and third quartile is indicated, and the whiskers are drawn down to the 10th percentile and up to the 90th.

C RPE1 cells were depleted for p53 in combination with the individual candidates. Cells were left untreated or irradiated with 4 Gy and incubated for 10 days after which the colonies were analyzed. Experiments were performed in triplicate and two independent times. Error bars represent 95% confidence interval (CI).

© 2019 The Authors

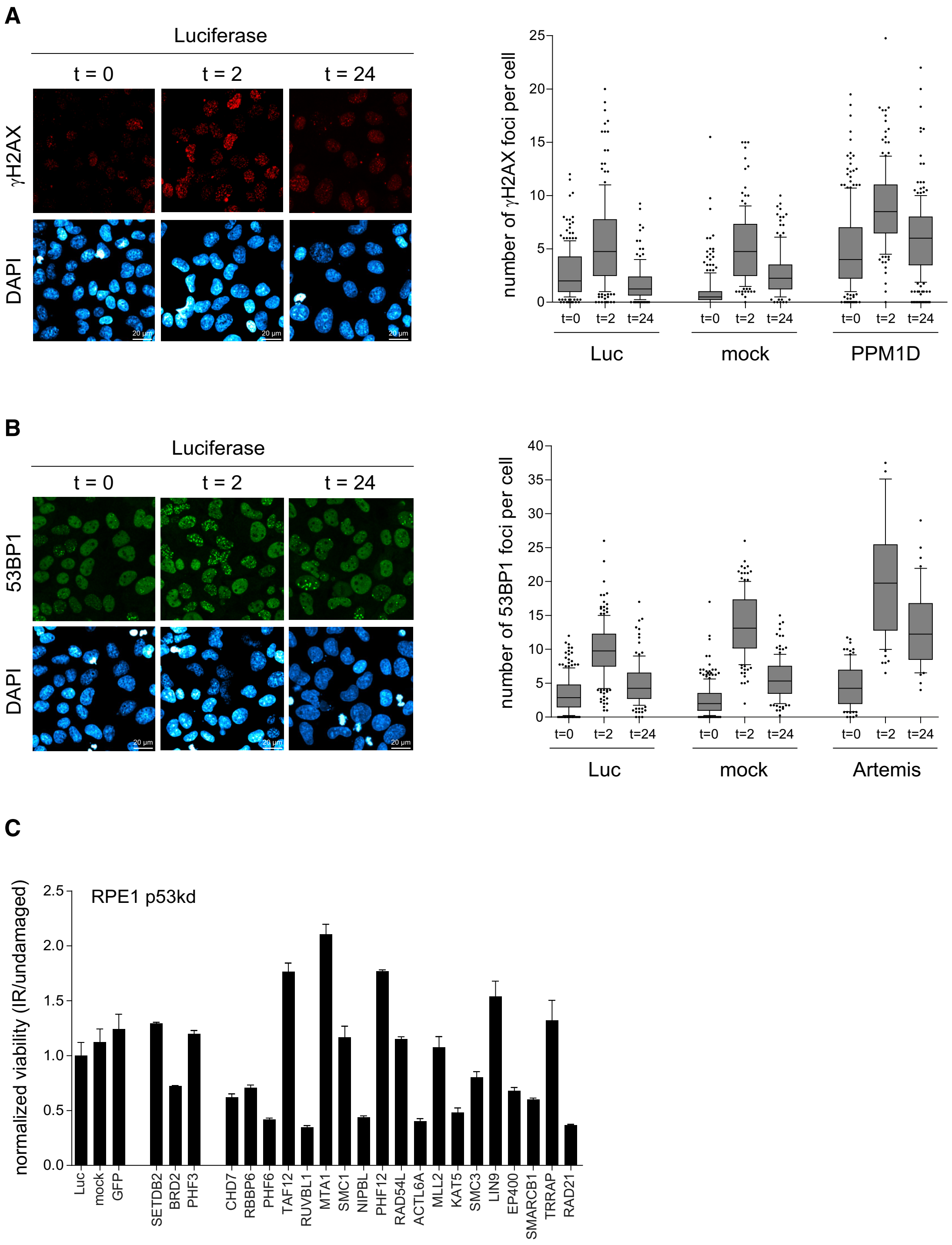

Figure EV1.

 © 2019 The Authors

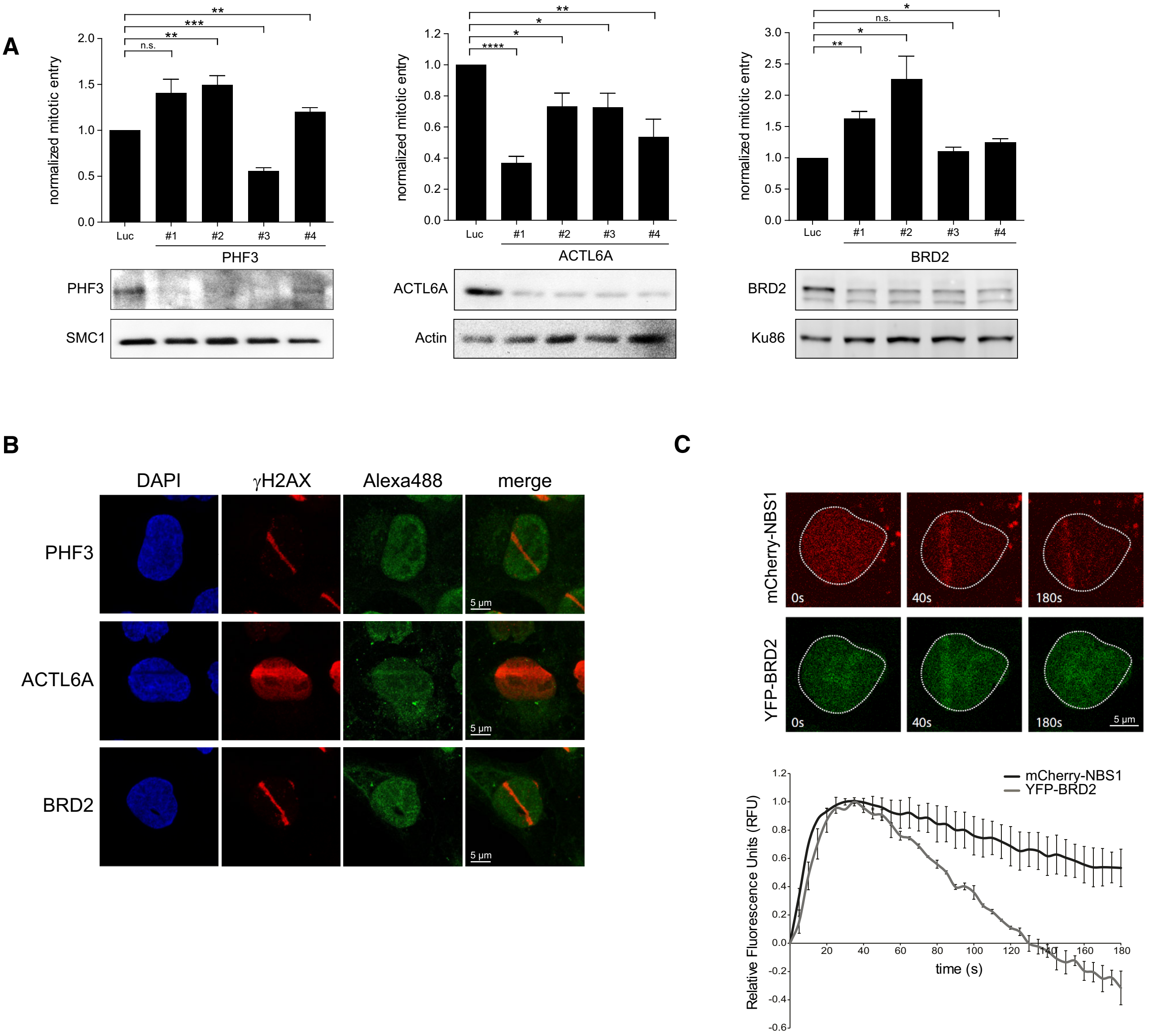

Figure EV2. Characterization of PHF3, ACTL6A, and BRD2 in G2 checkpoint recovery.

A U2OS cells were depleted for PHF3, BRD2, and ACTL6A and prepared for analysis by flow cytometry to determine checkpoint recovery (top panels) or lysed for Western blotting using the indicated antibodies (bottom panels). Error bars represent the SEM of three independent experiments. Statistical significance was determined using a two-tailed, unpaired *t*-test (**P* < 0.05, ***P* < 0.01, ****P* < 0.001, *****P* < 0.0001).

B U2OS cells were locally irradiated using a 405nm laser and fixed directly thereafter for immunofluorescence. Alexa 488 represents PHF3, ACTL6A, or BRD2 staining. Scale bar is 5 μm.

C U2OS cells expressing YPF-BRD2 and mCherry-NBS1 were locally irradiated and followed by time-lapse imaging. Graph shows the quantifications of at least 30 cells analyzed in three individual experiments, and error bars represent the SD. Scale bar is 5 μm.

© 2019 The Authors

Figure EV3. PHF6 knockout cells display a similar phenotype as cells depleted for PHF6.

A, B RPE1 cells were depleted of PHF6 using four different siRNA oligonucleotides. Checkpoint recovery after 5 Gy was analyzed by staining for MPM2 and flow cytometry (A), or cells were lysed and analyzed using Western blotting with the indicated antibodies (B). Error bars represent the SEM of three independent experiments. Statistical significance was determined using a two-tailed, unpaired *t*-test (***$P < 0.001$, ****$P < 0.0001$).

C U2OS WT and three different PHF6 knockout clones were lysed and analyzed by Western blotting with the indicated antibodies.

D U2OS WT and PHF6 knockout cells were irradiated at 2 Gy and fixed for immunofluorescence at 0, 1, and 16 h. Samples were stained with the indicated antibodies. Scale bar is 10 µm.

E Quantification of number of 53BP1 IRIF per cell of (D) in which the error bars represent the SD of three independent experiments. Statistical significance was determined using a two-tailed, unpaired *t*-test (**$P < 0.01$, ****$P < 0.0001$).

F U2OS cells were depleted of PHF6 with three different siRNA oligonucleotides. Cells were fixed, stained with propidium iodide, and analyzed by flow cytometry. Cell cycle distribution was determined using Flowlogic software. Error bars represent the SD of three independent experiments.

G U2OS WT and two different PHF6 knockout cell lines were fixed, stained with propidium iodide, and analyzed by flow cytometry. Cell cycle distribution was determined using Flowlogic software. Error bars represent the SD of three independent experiments.

© 2019 The Authors

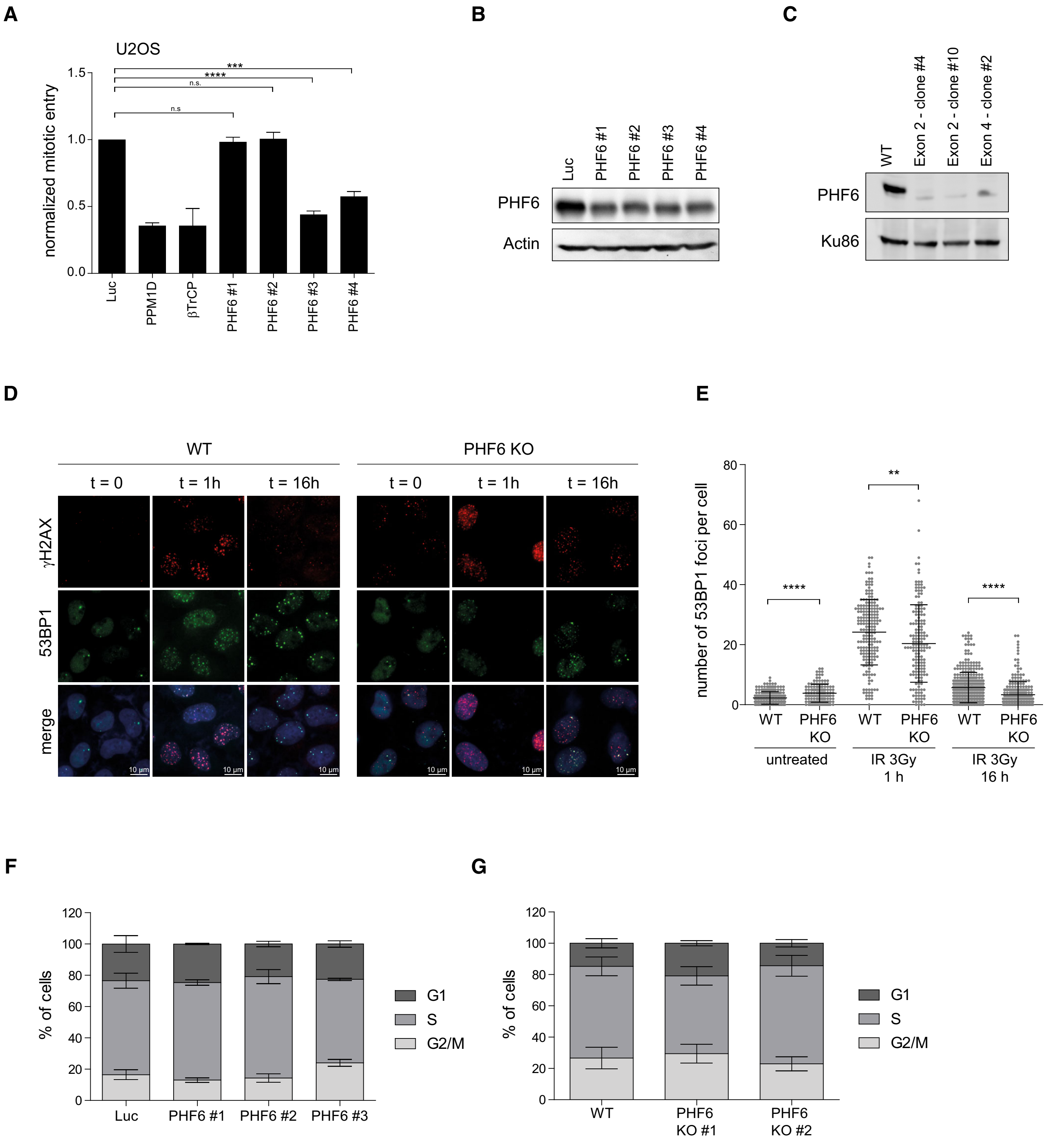
A
U2OS
normalized mitotic entry
1.5
1.0
0.5
0.0
***
****
n.s.
n.s
Luc
PPM1D
βTrCP
PHF6 #1
PHF6 #2
PHF6 #3
PHF6 #4
B
Luc
PHF6 #1
PHF6 #2
PHF6 #3
PHF6 #4
PHF6
Actin
C
WT
Exon 2 - clone #4
Exon 2 - clone #10
Exon 4 - clone #2
PHF6
Ku86
D
WT
PHF6 KO
t = 0
t = 1h
t = 16h
γH2AX
53BP1
merge
10 μm
E
number of 53BP1 foci per cell
80
60
40
20
0
****
**
****
WT
PHF6 KO
untreated
IR 3Gy 1 h
IR 3Gy 16 h
F
% of cells
120
100
80
60
40
20
0
Luc
PHF6 #1
PHF6 #2
PHF6 #3
G1
S
G2/M
G
% of cells
WT
PHF6 KO #1
PHF6 KO #2
G1
S
G2/M

Figure EV3.

Figure EV4. PHD domains required for checkpoint recovery function of PHF6.

A Graphical explanation of the PHD domains in PHF6 and the PHD1 and PHD2 deletion mutants (ΔPHD1 and ΔPHD2) generated for these studies.

B U2OS PHF6 knockout (KO) cells complemented with GFP-PHF6 wild type (GFP-PHF6 WT) or mutants lacking either the PHD1 (GFP-PHF6 ΔPHD1) or PHD2 (GFP-PHF6 ΔPHD2) domain were lysed and analyzed using Western blotting with the indicated antibodies.

C–E U2OS WT, PHF6 KO, or PHF6 KO cells complemented with GFP-PHF6 WT, GFP-PHF6 ΔPHD1, or GFP-PHF6 ΔPHD2 were irradiated using 3 Gy of IR and fixed after 1 h (C, D) or 24 h (E) for immunofluorescence. Presented is the number of γH2AX (C, E) or 53BP1 (D) foci per cell. Error bars represent the SD of three independent experiments. Statistical significance was determined using a two-tailed, unpaired *t*-test (*$P < 0.05$, **$P < 0.01$, ****$P < 0.0001$).

F U2OS WT, PHF6 KO, and PHF6 KO cells transfected with GFP-PHF6 WT, GFP-PHF6 ΔPHD1, or GFP-PHF6 ΔPHD2 were synchronized in G2, treated with etoposide (3 μM) for 1 h and then with nocodazole for 16 h. Subsequent recovery was analyzed by flow cytometry with MPM2 staining. Error bars represent the SEM of five independent experiments. Statistical significance was determined using a two-tailed, unpaired *t*-test (*$P < 0.05$, ****$P < 0.0001$).

© 2019 The Authors

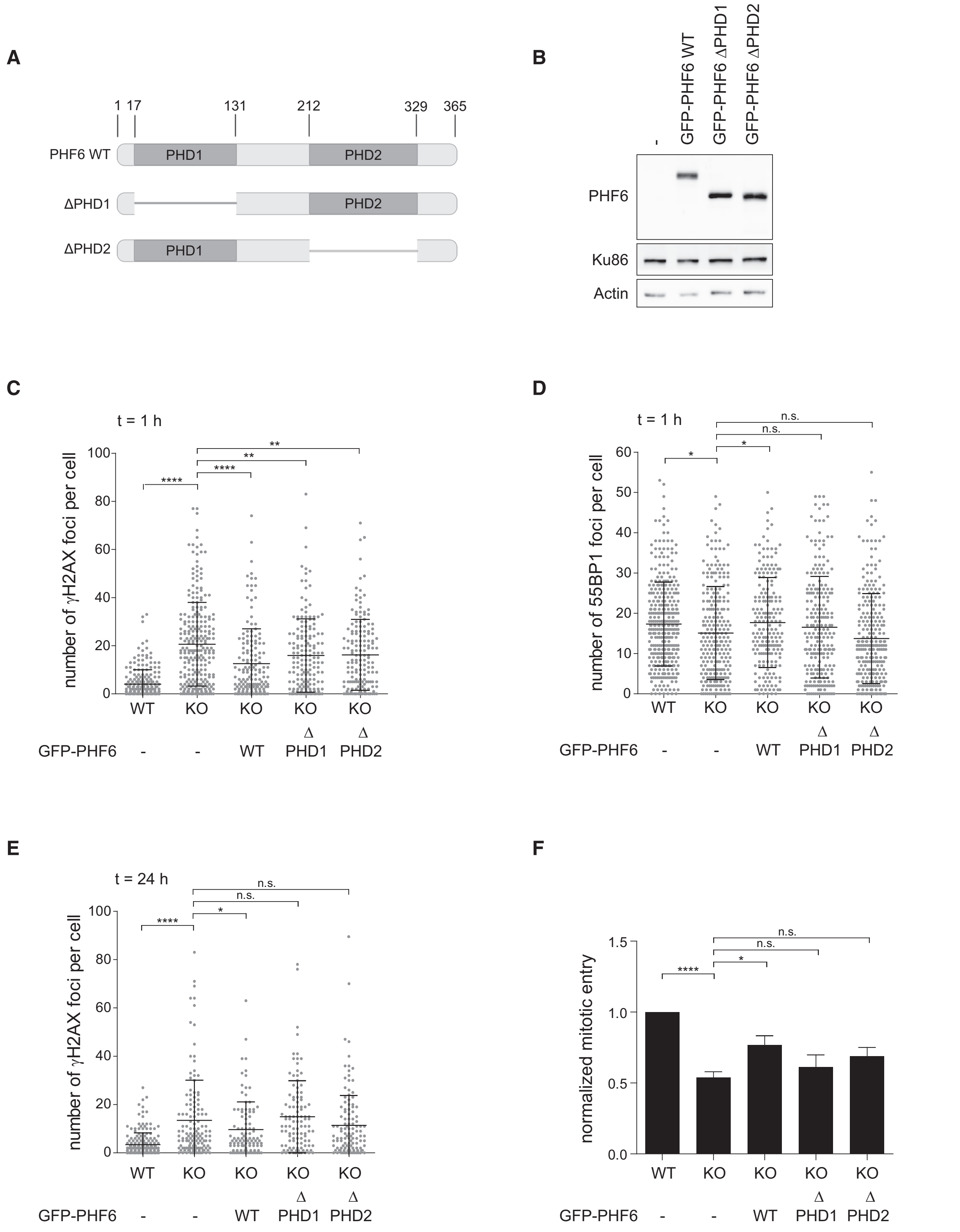
A
1 17 131 212 329 365
PHF6 WT PHD1 PHD2
ΔPHD1 PHD2
ΔPHD2 PHD1
B
-
GFP-PHF6 WT
GFP-PHF6 ΔPHD1
GFP-PHF6 ΔPHD2
PHF6
Ku86
Actin
C
t = 1 h
number of γH2AX foci per cell
WT KO KO KO KO
GFP-PHF6 - - WT ΔPHD1 ΔPHD2
D
t = 1 h
number of 55BP1 foci per cell
WT KO KO KO KO
GFP-PHF6 - - WT ΔPHD1 ΔPHD2
E
t = 24 h
number of γH2AX foci per cell
WT KO KO KO KO
GFP-PHF6 - - WT ΔPHD1 ΔPHD2
F
normalized mitotic entry
WT KO KO KO KO
GFP-PHF6 - - WT ΔPHD1 ΔPHD2

Figure EV4.

Figure EV5. PHF6 controls classical NHEJ.

A, B U2OS cells expressing GFP-PHF6 and mCherry-NBS1 were locally irradiated in the absence and presence of ATM and ATR inhibitors. Shown is the recruitment of GFP-PHF6 (A) or mCherry-NBS1 (B) to laser-induced breaks in time. At least 30 cells were analyzed in three individual experiments, and error bars represent the SD.

C U2OS-DR cells containing the I-SceI HR reporter were transfected with the indicated siRNA oligos and I-SceI. 48 h later, the cells were fixed and analyzed by flow cytometry. Represented is the relative repair efficiency as compared to the Luciferase control. Error bars represent the SEM of three independent experiments. Statistical significance was determined using a two-tailed, unpaired *t*-test (**$P < 0.01$, ***$P < 0.001$, ****$P < 0.0001$).

D U2OS cells depleted of PHF6 with three different siRNA oligonucleotides and U2OS WT and PHF6 knockout cells were lysed and analyzed using Western blotting with the indicated antibodies.

E U2OS WT and PHF6 knockout cells transfected with siRNA oligos against luciferase or XRCC4 were lysed and analyzed using Western blotting with the indicated antibodies. The remaining PHF6 signal in the PHF6 KO sample is likely due to a cross reaction at similar height as PHF6 (also see Fig EV3C).

F, G Distribution of the deletion sizes (F) or the extent of microhomology for the category simple deletions (G) obtained from independently obtained I-SceI repair events (described in Fig 4E) in the indicated samples.

 © 2019 The Authors

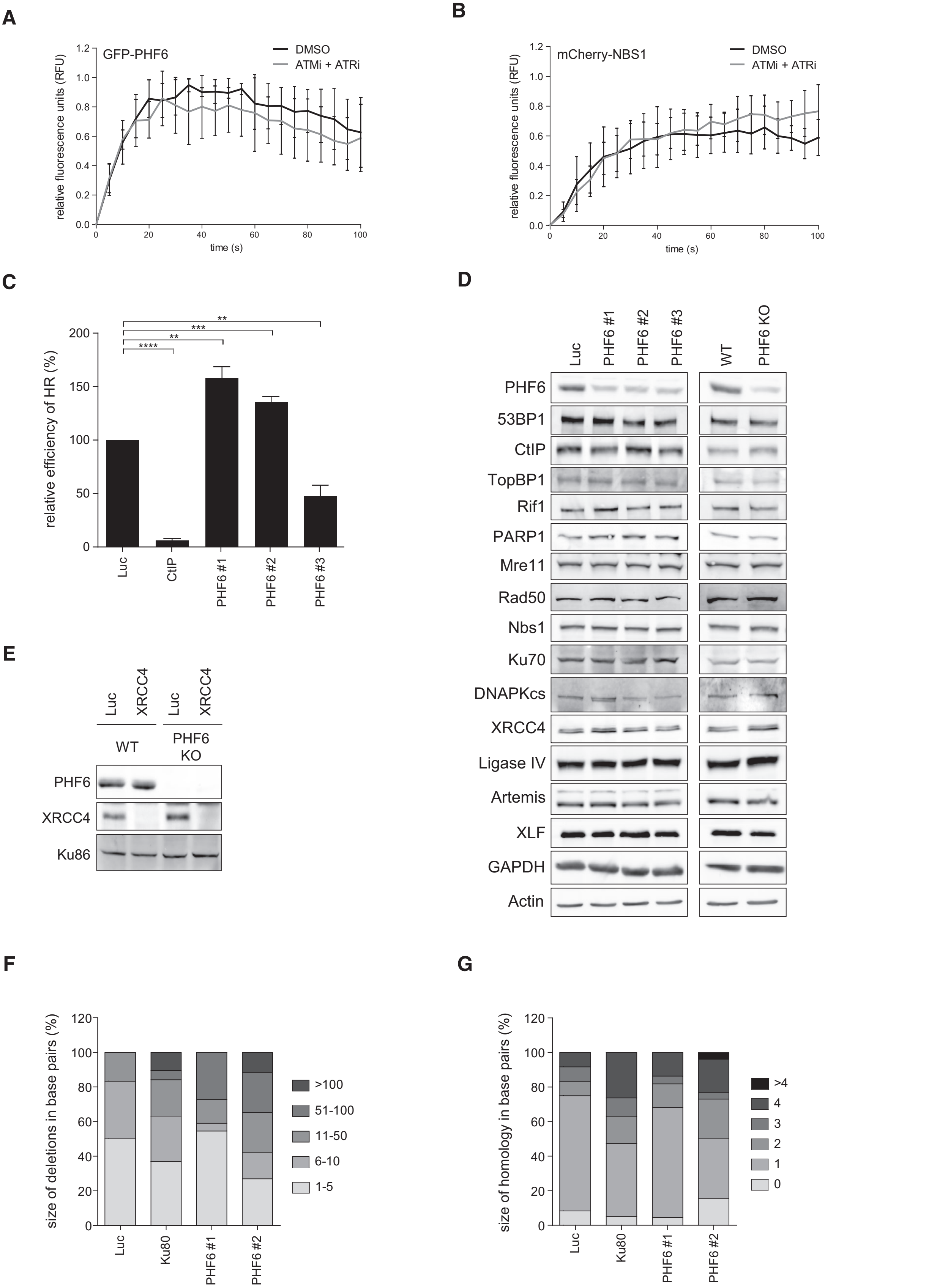
A
GFP-PHF6
DMSO
ATMi + ATRi
relative fluorescence units (RFU)
time (s)
B
mCherry-NBS1
DMSO
ATMi + ATRi
relative fluorescence units (RFU)
time (s)
C
relative efficiency of HR (%)
Luc
CtIP
PHF6 #1
PHF6 #2
PHF6 #3
D
Luc
PHF6 #1
PHF6 #2
PHF6 #3
WT
PHF6 KO
PHF6
53BP1
CtIP
TopBP1
Rif1
PARP1
Mre11
Rad50
Nbs1
Ku70
DNAPKcs
XRCC4
Ligase IV
Artemis
XLF
GAPDH
Actin
E
Luc
XRCC4
Luc
XRCC4
WT
PHF6 KO
PHF6
XRCC4
Ku86
F
size of deletions in base pairs (%)
>100
51-100
11-50
6-10
1-5
Luc
Ku80
PHF6 #1
PHF6 #2
G
size of homology in base pairs (%)
>4
4
3
2
1
0
Luc
Ku80
PHF6 #1
PHF6 #2

Figure EV5.
