# Supplementary material for: PHF6 promotes non‐homologous end joining and G2 checkpoint recovery
Source: EMBO Rep. 2019 Nov 29;21(1):e48460. doi: 10.15252/embr.201948460 (PMC6944915; doi:10.15252/embr.201948460)
Supplement: Supplementary file 2 — Table EV1 [file EMBR-21-e48460-s002.docx]

**Table EV1. Gene hits that influenced recovery.**

List of 22 gene hits that influenced G2 checkpoint recovery.

Increased recovery

| **Gene** |
| --- |
| SETDB2 |
| BRD2 |
| PHF3 |

Reduced recovery

| **Gene** |
| --- |
| CHD7 |
| RBBP6 |
| PHF6 |
| TAF12 |
| RUVBL1 |
| MTA1 |
| SMC1 |
| NIPBL |
| PHF12 |
| RAD54L |
| ACTL6A |
| MLL2 |
| KAT5 |
| SMC3 |
| LIN9 |
| EP400 |
| SMARCB1 |
| TRRAP |
| RAD21 |
